# Prolonged Systemic Inflammatory Response Syndrome Predicts Atrial Fibrillation After Cardiac Surgery

**DOI:** 10.1093/icvts/ivag081

**Published:** 2026-03-16

**Authors:** Emma Viikinkoski, Joonas Lehto, Arto Relander, Juho Jalkanen, Jarmo Gunn, Tuija Vasankari, Fausto Biancari, Juhani K E Airaksinen, Maija Hollmén, Tuomas O Kiviniemi

**Affiliations:** Heart Center, Turku University Hospital and University of Turku, FI-20521 Turku, Finland; Heart Center, Turku University Hospital and University of Turku, FI-20521 Turku, Finland; Heart Center, Turku University Hospital and University of Turku, FI-20521 Turku, Finland; MediCity Research Laboratory, Department of Microbiology and Immunology, InFLAMES Flagship, University of Turku, FI-20014 Turku, Finland; Heart Center, Turku University Hospital and University of Turku, FI-20521 Turku, Finland; Heart Center, Turku University Hospital and University of Turku, FI-20521 Turku, Finland; Department of Cardiovascular Surgery, Centro Cardiologico Monzino IRCCS, 20138 Milan, Italy; Heart Center, Turku University Hospital and University of Turku, FI-20521 Turku, Finland; MediCity Research Laboratory, Department of Microbiology and Immunology, InFLAMES Flagship, University of Turku, FI-20014 Turku, Finland; Heart Center, Turku University Hospital and University of Turku, FI-20521 Turku, Finland

**Keywords:** atrial fibrillation, cardiac surgery, cardiopulmonary bypass, transfusion, systemic inflammatory response syndrome

## Abstract

**Objectives:**

Cardiac surgery and the use of cardiopulmonary bypass (CPB) lead to short-lasting postoperative inflammatory response and some patients fail to adapt to the stress leading to a prolonged systemic inflammatory response syndrome (SIRS). We aimed to identify the risk factors for prolonged SIRS and whether this may affect the onset of short- and long-term postoperative atrial fibrillation (AF) after adult cardiac surgery patients.

**Methods:**

The CAREBANK biobank study consists of prospectively enrolled patients undergoing adult cardiac surgery from 2016 to 2021 with ongoing follow-up data. This substudy included patients operated on with or without the use of CPB.

**Results:**

Overall, 982 patients underwent cardiac surgery, 824 (84%) patients using CPB. Prolonged SIRS was observed in 62 (6.3%) patients. Transfusion of packed red blood cells (OR 1.9, 95%, confidence interval [CI] 1.1-3.5, *P* = .03), and the first postoperative day C-reactive protein level (OR 1.2, 95%, CI 1.0-1.3, per 10 units, *P* = .002) were associated with the development of prolonged SIRS in a multivariable analysis. Patients with prolonged SIRS had more adverse events during index hospitalization, mainly driven by the higher incidence of postoperative AF compared to non-SIRS patients (OR 2.4, 95%, CI, 1.4-4.0, *P* < .001). At 2 years, the incidence of post-discharge AF was higher compared with non-SIRS patients (hazard ratio 2.0, 95% CI, 1.1-3.6, *P* = .024).

**Conclusions:**

A subset of cardiac surgery patients demonstrates impaired adaptation to the perioperative inflammatory response, placing them at increased risk for AF both early after surgery and following discharge.

**Clinical registration number:**

NCT03444259

## GRAPHICAL ABSTRACT

**Figure:**
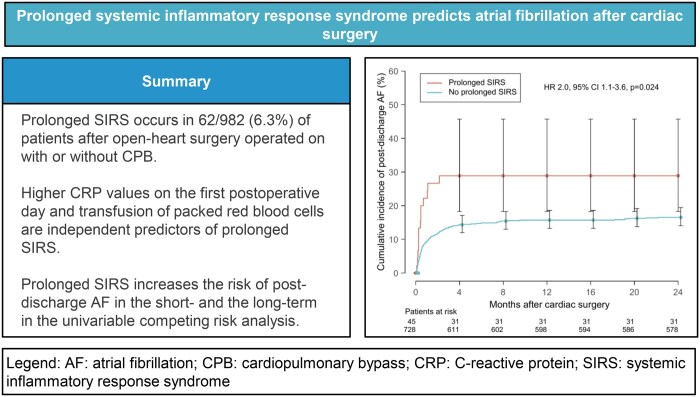

## INTRODUCTION

Postoperative atrial fibrillation (POAF) is a common, often transient phenomenon affecting one-third of patients undergoing cardiac surgery and may resolve within minutes to hours without intervention.^1^ The episodes of POAF usually occur 2-4 days after procedure prolonging the length of intensive care and hospitalization, increasing hospital treatment costs, and elevating the risk of long-term stroke, atrial fibrillation (AF), and mortality.^1–4^ The high occurrence of POAF after cardiac surgery involves a multitude of triggers affecting the homeostasis of the left atrium, such as hypoxia, increased levels of catecholamines, stretch related to the surgery itself, hypovolaemia, fluid resuscitation, and inflammation.^5–7^ In addition, pre-existing changes in atrial protein structure may predispose to POAF.^8^

Cardiopulmonary bypass (CPB) induces a release of fast-acting interleukins, especially in patients with hyperdynamic circulatory instability.^9–11^ Systemic inflammation increases oxidative stress, activates leucocyte expression, and degrades endothelial homeostasis regulators, all of which contribute to the pathogenesis of POAF.^11^^,^^12^ The use of statins, which have anti-inflammatory properties has not reduced the occurrence of POAF, but repeated dosage of glucocorticoids postoperatively have shown benefit in the reduction of AF.^13^^,^^14^ The majority of cardiac surgery patients encounter a drastic rise in cytokines perioperatively and develop postoperative systemic inflammation response.^15^ Interestingly, a subset of patients fails to adapt to the stress response within a few days and were recently identified as having cardiac surgery-associated prolonged systemic inflammatory response syndrome (SIRS), defined by fulfilling the clinical criteria of SIRS on 4 consecutive postoperative days (POD).^16^ Patients with prolonged SIRS have different inflammatory profiles preoperatively, a higher incidence of POAF, and worse short-term survival compared to those without. However, there is a lack of data on the impact of SIRS on long-term outcomes. It also remains unknown which perioperative factors are associated with the development of prolonged SIRS. These issues have been evaluated in the present prospective study.

## METHODS

The present study is based on the data from a prospective cohort of 1001 consecutive adult patients aged 18 years or older, who underwent cardiac surgery with or without the use of CPB from February 2016 to December 2021, at the Heart Center of the Turku University Hospital, Turku, Finland. Patients were participants in the CAREBANK biobank study (Cardiovascular Research Consortium—a Prospective Project to Identify Biomarkers of Morbidity and Mortality in Cardiovascular Interventional Patients), an ongoing Finnish prospective cohort study (ClinicalTrials.gov Identifier: NCT03444259). Detailed methods have been published in our previous study.^10^

Five patients with concomitant descending or abdominal aortic repair, 6 with cancelled procedures, 6 who died within the first 4 PODs, and 2 with missing data were excluded. After the exclusion, the study population includes 982 patients.

The study was approved by the Medical Ethics Committee of the Wellbeing Services County of Southwest Finland. The collection and storage of data and biological matter adhere to the World Medical Association Declaration of Taipei (2016) and were monitored by an independent third party. Written informed consent was obtained from the study subjects.

### Definitions

SIRS was diagnosed when 2 or more of the following were present: heart rate over 90 bpm, body temperature below 36 °C or over 38 °C, respiratory rate over 20/minute, low carbon dioxide level in arterial blood gas analysis (below 4.3 kPa), or an abnormal leucocyte count (<4 × 109/L or >12 × 109/L). Prolonged SIRS was defined as SIRS present on 4 consecutive PODs after surgery. Patients without prolonged SIRS were classified as non‑SIRS patients. Transfusion of packed red blood cells (RBC) adhered to strict transfusion protocol. Definitions of outcomes are provided in Supplementary Material (**Table S1**).

### Biomarkers

Cardiac troponin T (cTnT) and C-reactive protein (CRP) measurements were collected from index hospitalization blood samples during operation or in the morning time.

### Aims

The primary outcome of this study was AF occurring during the index hospitalization and the long-term period. Secondary outcomes were other events such as stroke, transient ischaemic attack (TIA), infection, and mortality during the index hospitalization and follow-up both individually and as a composite outcome.

### Statistical methods

The data were tested for normal distribution using the Shapiro-Wilk and Kolmogorov-Smirnov tests. Univariable logistic regression was performed to identify risk factors for prolonged SIRS and postoperative adverse outcomes. Multivariable logistic regression was performed by including variables of relevance with *P*-value <.05 in the univariable analyses. Univariable competing risk analysis with death as a competing event was conducted using the Fine-Gray subdistribution hazard model. A formal sample size calculation was not performed due to the observational nature of the study. The full statistical analyses are detailed in the Supplementary Material.

## RESULTS

### Patient demographics

Altogether, 824/982 patients underwent adult cardiac surgery with the use of CPB. The number of patients meeting the SIRS criteria on the first 4 PODs were 466 (47%), 638 (65%), 328 (33%), and 169 (17%), respectively. Prolonged SIRS was observed in 62 (6.3%) patients.

Patients with prolonged SIRS had decreased glomerular filtration rate (eGFR) at baseline and more often type 1 diabetes and hypertension (**Table 1**). Prolonged SIRS was observed more often in patients who were admitted for acute coronary syndrome requiring CABG and less often in patients undergoing elective mitral valve surgery (**Table 1** and **Table S2**). The use of calcium-channel blockers was more common in patients with prolonged SIRS (**Table S3**). Patients with prolonged SIRS had higher RBC transfusion need and lower haemoglobin levels during the first postoperative week compared to the non-SIRS patients.

**Table 1.** Baseline Demographics and Perioperative Characteristics of Patients With and Without Prolonged SIRS After Open-Heart Surgery and Univariable Logistic Regression Odds Ratios

	Prolonged SIRS	No prolonged SIRS	OR (95% CI)	*P*-value
	*n* = 62	*n* = 920		
Age (years)	71 (64-75)	67 (60-73)	1.2 (0.9-1.5) per 10 units	.13
BMI (kg/m^2^)	27 (25-32)	28 (25-31)	1.0 (0.9-1.1)	.85
Preoperative eGFR (mL/min/1.73 m^2^)	71 (53-87)	78 (64-91)	0.8 (0.7-1.0) per 10 units	.006
Chronic dialysis	2 (3.2%)	9 (1.0%)	3.4 (0.7-16.0)	.13
Male sex	52 (83.9%)	708 (76.5%)	1.6 (0.8-3.2)	.19
Treatment for diabetes type 2	15 (24.2%)	208 (22.6%)	1.1 (0.6-2.0)	.77
Treatment for diabetes type 1	6 (9.7%)	29 (3.2%)	3.3 (1.3-8.3)	.011
Treatment for hypertension	52 (83.9%)	631 (68.6%)	2.4 (1.2-4.8)	.014
Heart failure	11 (17.7%)	113 (12.3%)	1.5 (0.8-3.0)	.21
History of atrial fibrillation	17 (27.4%)	192 (20.9%)	1.4 (0.8-2.6)	.23
Chronic lung disease	12 (19.4%)	117 (12.7%)	1.6 (0.9-3.2)	.14
Active smoking	6 (9.7%)	125 (13.6%)	0.7 (0.3-1.6)	.38
Type of procedure				
AVR	19 (30.6%)	285 (31.0%)	1.0 (0.6-1.7)	.96
CABG	43 (69.4%)	512 (55.7%)	1.8 (1.0-3.1)	.037
Any mitral valve procedure	4 (6.5%)	162 (17.6%)	0.3 (0.1-0.9)	.031
Maze procedure	1 (1.6%)	24 (2.6%)	0.6 (0.1-4.6)	.63
Pericardiectomy	0 (0%)	5 (0.5%)	NS	NS
LAA closure	8 (12.9%)	135 (14.7%)	0.9 (0.4-1.9)	.7
Ascending aorta	4 (6.5%)	100 (10.9%)	0.6 (0.2-1.6)	.28
Operation specifics				
CPB	51 (82.3%)	773 (84.0%)	0.9 (0.5-1.7)	.72
EuroSCORE II (%)	1.91 (1.37-2.98)	1.48 (0.97-2.56)	1.1 (1.0 -1.2)	.19
ACS	30 (48.4%)	275 (29.9%)	1.8 (1.0-3.3)	.038
CPB time (minutes)	122 (107-148)	119 (102-145)	1.0 (1.0-1.1) per 10 units	.77
Aortic cross-clamping time (minutes)	99 (26)	90 (76-111)	1.0 (1.0-1.1) per 10 units	.41
Blood products	35 (56.5%)	414 (45.0%)	1.6 (0.9-2.7)	.08
RBC transfusion	30 (48.4%)	304 (33.0%)	1.9 (1.1-3.2)	.015
RBC units/patient	2.0 (1.8-4.3)	2.0 (2.0-4.0)	0.9 (0.8-1.1)	.36
Lowest Hb postoperatively (g/L)	111 (13)	114 (102-125)	0.9 (0.8-1.1) per 10 units	.29
Lowest Hb during the first postoperative week (g/L)	85 (78-95)	93 (83-104)	0.6 (0.4-0.8) per 10 units	<.001

Preoperative eGFR was calculated using CKD-EPI equation.

Abbreviations: ACS, acute coronary syndrome; AVR, aortic valve replacement; BMI, body mass index; CABG, coronary artery bypass grafting; CPB, cardiopulmonary bypass; eGFR, estimated glomerular filtration rate; Hb, haemoglobin; NS, not significant; RBC, packed red blood cell; SIRS, systemic inflammatory response syndrome.

### Clinical biomarkers

**Figure 1.**
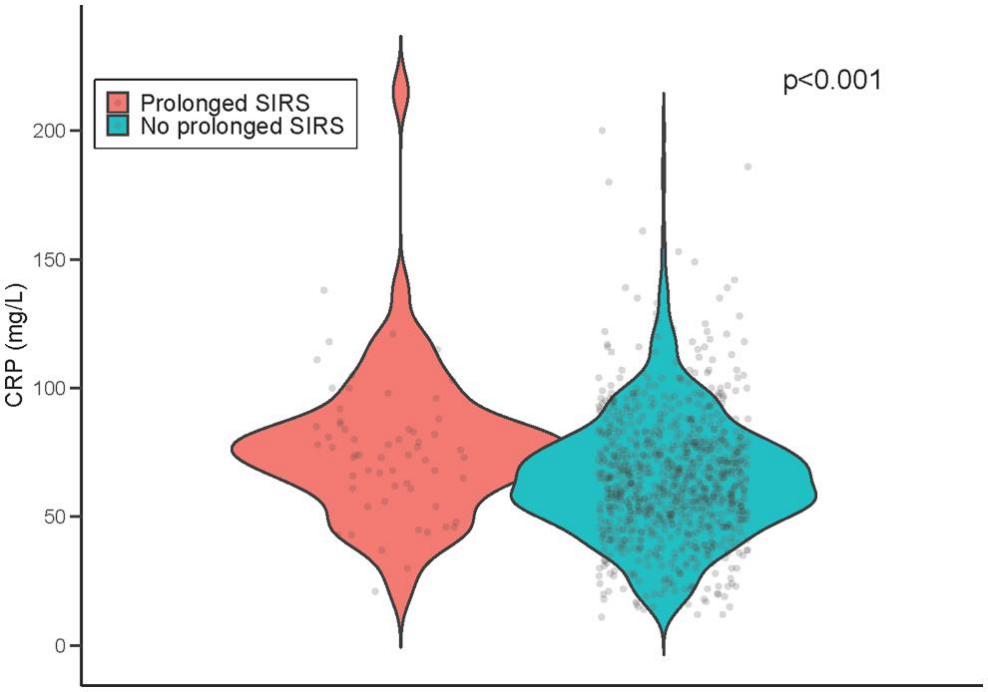
Violin Plot of the First Postoperative Day CRP Distribution in Patients With and Without Prolonged SIRS (*n* = 965).

**Figure 2.**
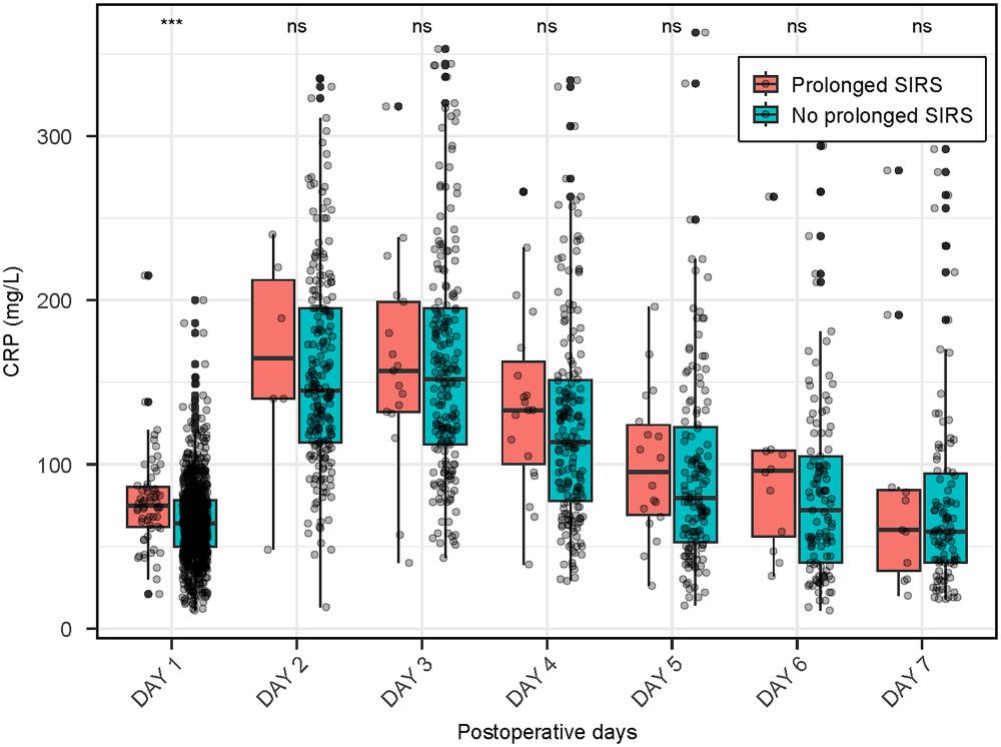
CRP Values During the First Postoperative Week in Patients with Prolonged Systemic Inflammatory Response Syndrome (SIRS) and Without. CRP was measured once for 958 patients and at least twice for 501 patients during the first postoperative week. *** P<0.05.

Higher CRP levels on the first POD were associated with prolonged SIRS (median 75 mg/L (IQR 61-87 mg/L) vs non-SIRS group 64 mg/L (IQR 50-78 mg/L), OR 1.2, 95% confidence interval [CI] 1.1-1.3, per 10 units *P* < .001) (**Figure 1**). In addition, patients with prolonged SIRS had significantly higher CRP levels during the first postoperative week (median 115 mg/L, IQR 73-164 mg/L) compared with non-SIRS patients (median 82 mg/L, IQR 58-129 mg/L, *P* = .002), although there was no difference in the daily concentrations after the first POD (**Figure 2**).

The highest measured cTnT levels were similar between the study groups during operation, on the first POD and during the first postoperative week (**Table S4**).

In a multivariable logistic regression model including significant clinical biomarkers and baseline and perioperative risk factors from the univariable analysis, RBC transfusion (OR 1.9, 95% CI 1.1-3.5, *P* = .03) and higher CRP on the first POD (OR 1.2, 95% CI 1.0-1.3, per 10 units, *P* = .002) were independently associated with prolonged SIRS (**Table S5**).

### In-hospital adverse outcomes

The occurrence of POAF was higher with prolonged SIRS patients than with non-SIRS patients (62.2% vs 37.0%, OR 2.8, 95% CI 1.5-5.2, *P* = .001) (**Table 2**). The median time for POAF diagnosis was 2 days (IQR 1-4 days vs IQR 2-4 days, respectively, *P* = .23). Overall, patients with prolonged SIRS had significantly higher rates of composite adverse outcomes (OR 2.4, 95% CI 1.4-3.9, *P* = .001), driven by higher incidences of POAF and strokes, they underwent more often cardioversion, and had higher risk of persisting AF at discharge. The occurrences of postoperative infection, acute de novo dialysis, or mortality did not differ between the groups.

**Table 2.** Adverse Outcomes of Patients With and Without Prolonged SIRS After Open-Heart Surgery

	Prolonged SIRS	No prolonged SIRS	OR (95% CI)	*P*-value
	*n* = 62	*n* = 920	Prolonged SIRS as a risk factor	
In-hospital				
Any adverse event	33 (53.2%)	300 (32.6%)	2.4 (1.4-3.9)	.001
POAF^a^	28 (62.2%)	268 (37.0%)	2.8 (1.5-5.2)	.001
POAF after PODs 1-4^a^	4 (19.0%)	50 (9.9%)	2.2 (0.7-6.6)	.18
Cardioversion^a^	4 (8.9%)	19 (2.6%)	3.6 (1.2-11.2)	.025
Stroke	4 (6.5%)	18 (2.0%)	3.5 (1.1-10.5)	.029
TIA	0 (0%)	2 (0.2%)	NS	NS
Postoperative pneumonia	3 (4.8%)	20 (2.2%)	2.3 (0.7-7.9)	.19
DSWI or mediastinitis	1 (1.6%)	9 (1.0%)	1.6 (0.2-13.3)	.63
Acute *de novo* dialysis	0 (0%)	6 (0.7%)	NS	NS
Death	1 (1.6%)	7 (0.8%)	2.1 (0.3-17.7)	.48
Length of hospital stay (days)	9 (8-12)	8 (7-10)	1.0 (1.0-1.1)	.16
Heart rhythm at discharge^a^				
Sinus rhythm	35 (77.8%)	642 (88.6%)	0.5 (0.2-0.9)	.035
AF or flutter	9 (20.0%)	58 (8.0%)	2.9 (1.3-6.3)	.008
Other^b^	1 (2.2%)	22 (3.0%)	NS	NS
90-day follow-up				
Any adverse events	18 (29.0%)	141 (15.3%)	2.3 (1.3-4.0)	.006
Post-discharge AF^a^	13 (28.9%)	99 (13.6%)	2.6 (1.3-5.1)	.006
MACCE	5 (8.1%)	46 (5.0%)	1.7 (0.6-4.4)	.3
Myocardial infarction	0 (0%)	2 (0.2%)	NS	NS
Stroke	4 (6.5%)	25 (2.7%)	2.5 (0.8-7.3)	.1
TIA	0 (0%)	7 (3.2%)	NS	NS
All-cause mortality	3 (4.8%)	15 (1.6%)	3.1 (0.9-10.9)	.08
1-year follow-up				
Any adverse events	20 (32.3%)	175 (19.0%)	2.0 (1.2-3.5)	.013
Post-discharge AF^a^	13 (28.9%)	117 (16.1%)	2.1 (1.1-4.2)	.029
Beta blockers^a^	39 (86.7%)	558 (76.6%)	2.0 (0.8-4.8)	.13
Permanent anticoagulation^a^	10 (22.2%)	72 (9.9%)	2.6 (1.2-5.5)	.012
MACCE	7 (11.3%)	69 (7.5%)	1.6 (0.7-3.6)	.28
Myocardial infarction	0 (0%)	4 (0.4%)	NS	NS
Stroke	4 (6.5%)	33 (3.6%)	1.9 (0.6-5.4)	.26
TIA	1 (1.6%)	12 (1.3%)	1.2 (0.2-9.7)	.84
All-cause mortality	4 (6.5%)	25 (2.7%)	2.5 (0.8-7.3)	.1

Univariable logistic regression analysis shows prolonged SIRS as a risk factor for set outcome.

Any adverse event during index hospitalization: a composite variable of POAF, stroke, TIA, postoperative pneumonia, DSWI/mediastinitis, *de novo* dialysis, and death. Any adverse events at follow-up: a composite variable of post-discharge AF and MACCE.

a Preoperative atrial fibrillation (*n* = 209) excluded.

b Junctional rhythm or pacemaker.

Abbreviations: AF, atrial fibrillation; DSWI, deep sternal wound infection; MACCE, major adverse cardiovascular and cerebrovascular event s (all-cause mortality, stroke, or myocardial infarction); NS, not significant; POAF, postoperative atrial fibrillation; POD, postoperative day; SIRS, systemic inflammatory response syndrome; TIA, transient ischaemic attack.

### Follow-up

**Figure 3.**
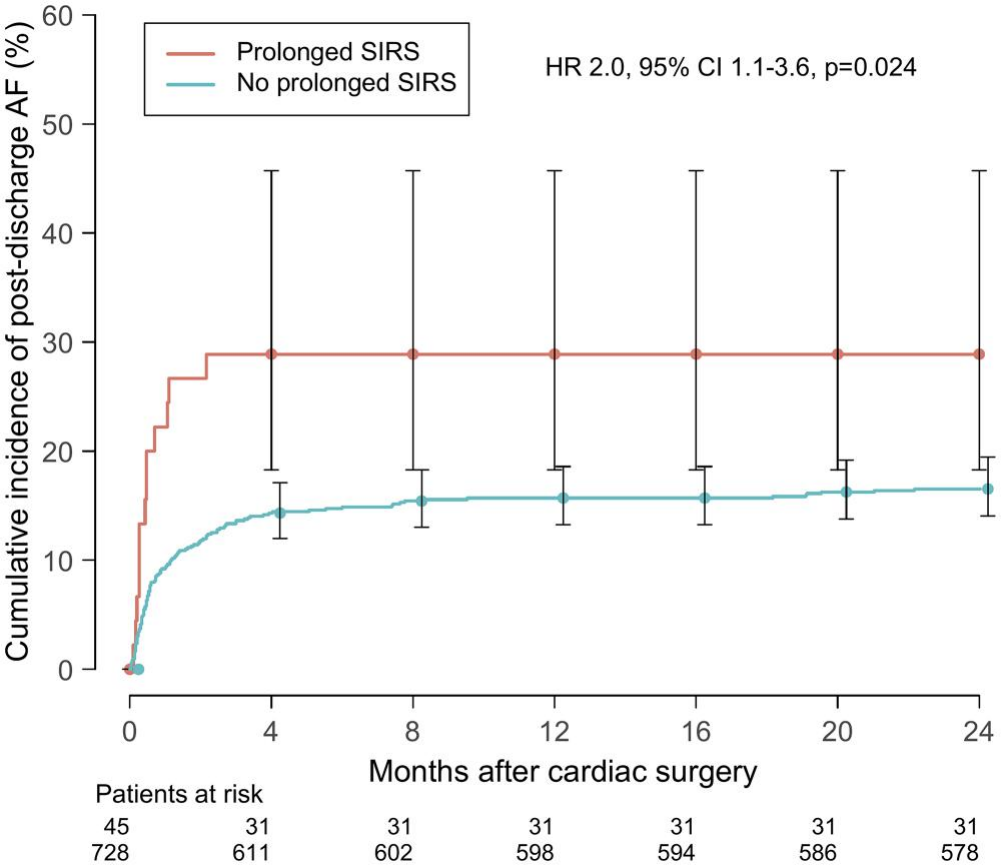
Cumulative Incidence of Post-Discharge Atrial Fibrillation (AF) in Patients With and Without Prolonged SIRS. Patients with preoperative AF were excluded (*n* = 209). Hazard ratio (HR) obtained from the fine-gray model describes the probability of AF occurring over 24 months and was complete for 98.7% of patients.

The incidence of post-discharge AF was 2-fold higher in patients with prolonged SIRS during all follow-up time-points (**Table 2**). Over the 2-year follow-up after index hospitalization, prolonged SIRS was a significant risk factor for AF (hazard ratio 2.0, 95% CI 1.1-3.6, *P* = .024, **Figure 3**), irrespective of the use of CPB (**Figure S1**). In addition, a multivariable logistic regression model including significant baseline and perioperative risk factors from the univariable analysis identified prolonged SIRS (OR 2.4, 95% CI 1.2-4.8), lower eGFR (OR 1.02, 95% 1.01-1.04), operation on mitral valve (OR 3.8, 95% CI 2.2-6.5), on ascending aorta (OR 1.9, 95% CI 1.0-3.4), or AVR (OR 2.5, 95% CI 1.6-3.9) as independent risk factors for AF during 2-year follow-up (**Table S6**). Otherwise, the risks of major adverse cardiovascular and cerebrovascular events were similar between the groups in the whole cohort.

## DISCUSSION

### Key findings

In this large prospective cohort of patients undergoing cardiac surgery, RBC transfusion and elevated CRP levels on the first POD were independent risk factors for prolonged SIRS—a persisting inflammatory state during hospitalization. The study confirms that a subset of patients fails to adapt to postoperative inflammation and 1 in 16 patients developing prolonged SIRS have a higher risk for in-hospital adverse outcomes and AF during follow-up. We propose that prolonged SIRS is an independent condition of inadequate adaptation to systemic inflammation after cardiac surgery, can be identified with SIRS criteria, and should be diagnosed to identify high-risk patients susceptible to AF.

### Prolonged inflammation and POAF

The occurrence of both symptomatic and asymptomatic POAF after cardiac surgery affects ∼20%-40% of patients and POAF is a risk factor for recurring AF after discharge.^17^^,^^18^ Inflammation during and after cardiac surgery causes atrial structural and functional changes that predispose to the development of AF.^5–7^^,^^11^^,^^12^ Elevated baseline CRP levels have been shown to associate with the development of AF in non-operative patients.^19^ Olesen et al^18^ found that higher CRP on the fourth POD increased the risk of POAF by 30% compared to lower CRP levels. In comparison, Ahlsson et al^20^ observed no association between POAF and elevated CRP levels on the third POD after cardiac surgery.

In the study, early CRP elevation predicted prolonged SIRS, suggesting that CRP initially signals inadequate adaption to systemic inflammation, followed by an increased risk for POAF. Moreover, patients with prolonged SIRS had higher CRP elevation during the first postoperative week compared with non-SIRS patients. In line with the findings, Chen et al^21^ observed that elevated systemic immune-inflammation index postoperatively associated with a higher occurrence of POAF following cardiac surgery. The use of minimally invasive extracorporeal circulation does not weaken attenuated inflammation compared to conventional CPB.^22^ Although higher CRP levels can indicate postoperative infection,^23^ rate of infections did not differ between the study groups.

Substantial variations in cTnT release are consistent with previous findings that individual troponin levels are influenced by multiple factors, including CPB, perioperative tachyarrhythmias, and postprocedural myocardial injury.^24^^,^^25^

In the study, transfusion independently associated with prolonged SIRS while baseline characteristics, drugs, the type and urgency of operation, or use of CBP did not. RBC transfusion hinders adaption to hypoxia through inhibiting hypoxia-inducible factor 1-alfa (HIF-1α) and greater transfusion need is associated with POAF, stroke, and reduced long-term survival.^10^^,^^26^^,^^27^ Observed risk factors for POAF range from advanced age, male sex, ethnicity, previous history of AF, chronic obstructive lung and cardiovascular diseases and drug treatment to use of CPB, atrial and valvular surgery, and RBC transfusion.^1^^,^^28^^,^^29^ Prolonged SIRS, induced in part by transfusion, might uniquely contribute to atrial remodelling and subsequent AF susceptibility.

### Prolonged inflammation and post-discharge period

The study showed double the risk of short- and long-term AF in the prolonged SIRS population. AF after major cardiac surgery is a risk for short- and long-term stroke, cardiovascular events, and reduced long-term survival.^3^^,^^4^^,^^30^

Although the risk of in-hospital stroke was higher in the prolonged SIRS group, the incidence of stroke during follow-up was too low to conclude whether prolonged SIRS, AF, anticoagulation therapy, or operation type played a role. The absolute risk of long-term stroke remains lower than the risk of short-term stroke after cardiac surgery,^31^ that can explain why the study found no difference between the study groups during long-term follow-up.

Systematic ECGs might be warranted for patients with prolonged SIRS to identify post-discharge AF during long-term follow-up. Future research on glucocorticoid preventative effect on prolonged SIRS is required.^13^^,^^14^

### Strengths and limitations

The study has many strengths. The large, prospectively enrolled patient cohort represents all eligible patients during the study period reducing sampling bias and allows to observe event rates to reflect the clinical population accurately. Multivariable models and detailed data analysis lessen confounding. The incidence of prolonged SIRS was unknown at the time of study planning and the current work represents a post hoc analysis, why no formal a priori sample size or power calculation was feasible. However, the study cohort includes detailed and consistent clinical phenotyping of 1001 patients—a sample size that provides a robust foundation for identifying clinically meaningful subgroups, including patients with prolonged SIRS, and supports the reliability of the observed associations. The findings of a single-centre study might limit the generalization of the results. Naturally, patients who chose not to participate might cause selection and attrition bias.

## CONCLUSION

The findings of this study suggest that a subset of patients undergoing cardiac surgery exhibit an abnormal adaptation to the significant inflammatory response caused by the surgery, CPB, and perioperative care, making them prone to developing AF both during the early postoperative period as well as after hospitalization. Further studies are needed to identify and address the factors contributing to this abnormal response.

## Supplementary Material

ivag081_Supplementary_Data

## Data Availability

The data underlying this article will be shared on reasonable request to the corresponding author.

## ABBREVIATIONS

AVR: Aortic valve replacement
BMI: Body mass index
CABG: Coronary artery bypass grafting
CPB: Cardiopulmonary bypass
CRP: C-reactive protein
cTnT: Cardiac troponin T
eGFR: Estimated glomerular filtration rate
EuroSCORE II: European System for Cardiac Operative Risk Evaluation II
LAA: Left atrial appendage
MACCE: Major adverse cardiovascular and cerebrovascular events
POAF: Postoperative atrial fibrillation
POD: Postoperative day
RBC: Red blood cell
SIRS: Systemic inflammatory response syndrome
TIA: Transient ischaemic attack
