# Supplementary material for: Prolonged Systemic Inflammatory Response Syndrome Predicts Atrial Fibrillation After Cardiac Surgery
Source: Interdiscip Cardiovasc Thorac Surg. 2026 Mar 16;41(3):ivag081. doi: 10.1093/icvts/ivag081 (PMC13025084; doi:10.1093/icvts/ivag081)

**Supplementary material**

**Statistical analysis**

**Supplementary Table 1. Supplementary Table 1.** Definitions of the outcomes.

**Supplementary Table 2.** A detailed list of baseline demographics and perioperative characteristics of patients with and without prolonged SIRS after open-heart surgery and univariable logistic regression odds ratios.

**Supplementary Table 3.** Preoperative medication in patients with and without prolonged SIRS.

**Supplementary Table 4.** The highest measured cTnT levels were similar between patients with and without prolonged SIRS during operation, the first postoperative day and during the first postoperative week.

**Supplementary Table 5.** Significant clinical biomarkers and baseline and perioperative risk factors from the univariable analysis included in the multivariable logistic regression model to assess association with prolonged SIRS.

**Supplementary Table 6.** Significant baseline and perioperative risk factors from the univariable analysis included in the multivariable logistic regression model to assess association with post-discharge atrial fibrillation (AF) during 2-year follow-up. Patients with preoperative atrial fibrillation (AF) (n=209) were not included.

**Supplementary Figure 1.** Cumulative incidence of post-discharge atrial fibrillation (AF) in patients with and without prolonged SIRS operated with cardiopulmonary bypass (n=824). Patients with preoperative AF were excluded (n=209). Hazard ratio (HR) obtained from the Fine-Gray model describes the probability of AF occurring over 24 months.

***Statistical analysis***

Continuous variables are reported as mean ± standard deviation (SD) if normally distributed and as median with the 25^th^ - 75^th^ percentiles as appropriate. Categorical variables are reported as counts and percentages. The data were tested for normal distribution using the Shapiro-Wilk and Kolmogorov-Smirnov tests. Univariable logistic regression was performed to identify risk factors for prolonged SIRS and postoperative outcomes. Multivariable logistic regression was performed by including variables of relevance with p-value <0.05 in the univariable analyses. A stepwise, backward approach was used. Univariable competing risk analysis with death as a competing event was conducted using the Fine-Gray subdistribution hazard model to estimate the association between prolonged SIRS and post-discharge AF. A formal sample size calculation was not performed due to the observational nature of the study. Statistical analyses were performed using IBM SPSS Statistics version 29 (SPSS Inc., Chicago, IL, USA) and R statistics software version 4.4.2 (R Foundation for Statistical Computing, Vienna Austria).

**Supplementary Table 1.** Definitions of the outcomes.

| **Outcomes** |  |
| --- | --- |
| Ischemic stroke and transient ischemic attack | Ischemic stroke was defined as a permanent focal neurological deficit adjudicated by a neurologist and confirmed via computed tomography or magnetic resonance imaging. Transient ischemic attack (TIA) was defined as a transient episode of neurological dysfunction caused by focal brain, spinal cord, or retinal ischemia, without acute infarction and complete resolve of symptoms within 24 hours. Only stroke and TIA events diagnosed by the treating neurologist or physician were considered in the present study. |
| Myocardial infarction | Myocardial infarction was defined as permanent cardiomyocyte injury in the setting of myocardial ischemia characterized with ST-segment changes on ECG with acute chest pain and cardiac troponin elevation. |
| Post-discharge atrial fibrillation | Post-discharge atrial fibrillation refers to a recurring paroxysmal or persistent atrial fibrillation (AF) in patients without history of preoperative paroxysmal or persistent AF or flutter. The diagnosis of post-discharge AF is irrespective of the occurrences of postoperative atrial fibrillation episodes during index hospitalization. Post-discharge atrial fibrillation was confirmed by 12-lead electrocardiogram (ECG) recording or continuous ECG monitoring of an episode lasting at least 5 minutes after index hospitalization. |
| Postoperative atrial fibrillation | Postoperative atrial fibrillation (POAF) refers to episodes of tachyarrhythmia during index hospitalization that may resolve within minutes or hours. POAF were confirmed by 12-lead electrocardiogram (ECG) recording or continuous ECG monitoring of an episode postoperatively during index hospitalization. |
| Postoperative mediastinitis  and pneumonia | Postoperative mediastinitis and pneumonia were defined as symptoms of infection, fever, malaise, elevation of C-reactive protein (CRP) or leukocyte count, and/or imaging finding consistent with infection at the mediastinal or pulmonary area and without signs of alternative diagnoses with similar presentation. |
| Reoperation for bleeding | Reoperation for bleeding was defined as a bleeding that required control through a resternotomy. |

**Supplementary Table 2.** A detailed list of baseline demographics and perioperative characteristics of patients with and without prolonged SIRS after open-heart surgery and univariable logistic regression odds ratios.

|  | | **Prolonged SIRS**  **n = 62** | | **No prolonged SIRS**  **n = 920** | **OR (95% CI)** | **P-value** |
| --- | --- | --- | --- | --- | --- | --- |
|  | |  |  |  |  |  |
| Age (years) | | | 71 (64-75) | 67 (60-73) | 1.2 (0.9-1.5) per 10 units | 0.13 |
| BMI (kg/m^2^) | | 27 (25-32) | | 28 (25-31) | 1.0 (0.9-1.1) | 0.85 |
| Preoperative eGFR (ml/min/1.73 m^2^) | | 71 (53-87) | | 78 (64-91) | 0.8 (0.7-1.0) per 10 units | 0.006 |
| Chronic dialysis | | 2 (3.2%) | | 9 (1.0%) | 3.4 (0.7-16.0) | 0.13 |
| Preoperative Hb (g/L) | | 136 (14) | | 139 (128-148) | 0.9 (0.8-1.1) per 10 units | 0.42 |
| Male sex | | 52 (83.9%) | | 708 (76.5%) | 1.6 (0.8-3.2) | 0.19 |
| Treatment for dyslipidemia | | 47 (75.8%) | | 613 (66.6%) | 1.6 (0.9-2.9) | 0.14 |
| Treatment for diabetes | | 21 (33.9%) | | 242 (26.3%) | 1.4 (0.8-2.5) | 0.2 |
| Type 2 | | 15 (24.2%) | | 208 (22.6%) | 1.1 (0.6-2.0) | 0.77 |
| Type 1 | | 6 (9.7%) | | 29 (3.2%) | 3.3 (1.3-8.3) | 0.011 |
| Insulin treatment | | 11 (17.7%) | | 100 (10.9%) | 1.8 (0.9-3.5) | 0.1 |
| Treatment for hypertension | | 52 (83.9%) | | 631 (68.6%) | 2.4 (1.2-4.8) | 0.014 |
| Heart failure | | 11 (17.7%) | | 113 (12.3%) | 1.5 (0.8-3.0) | 0.21 |
| History of atrial fibrillation | | 17 (27.4%) | | 192 (20.9%) | 1.4 (0.8-2.6) | 0.23 |
| History of coronary artery disease | | 37 (59.7%) | | 486 (52.8%) | 1.3 (0.8-2.2) | 0.3 |
| Previous myocardial infarction | | 9 (14.5%) | | 133 (14.5%) | 1.0 (0.5-2.1) | 0.99 |
| Recent myocardial infarction | | 11 (17.7%) | | 129 (14.0%) | 1.3 (0.7-2.6) | 0.42 |
| Prior cardiac procedure | | 6 (9.7%) | | 152 (16.5%) | 0.5 (0.2-1.3) | 0.16 |
| PCI | | 6 (9.7%) | | 121 (13.2%) | 0.7 (0.3-1.7) | 0.43 |
| CABG | | 0 (0%) | | 8 (0.9%) | NS | NS |
| Aortic or mitral valve prosthesis | | 0 (0%) | | 27 (2.9%) | NS | NS |
| TAVI | | 0 (0%) | | 3 (0.3%) | NS | NS |
| Prior stroke | | 4 (6.5%) | | 77 (8.4%) | 0.8 (0.3-2.1) | 0.6 |
| Extracardiac arteriopathy | | 5 (8.1%) | | 59 (6.4%) | 1.3 (0.5-3.3) | 0.61 |
| Carotid artery disease | | 2 (3.2%) | | 18 (2.0%) | 1.7 (0.4-7.4) | 0.5 |
| Peripheral artery disease | | 5 (8.1%) | | 48 (5.2%) | 1.6 (0.6-4.2) | 0.34 |
| Previous endocarditis | | 0 (0%) | | 7 (0.8%) | NS | NS |
| Active endocarditis | | 0 (0%) | | 6 (0.6%) | NS | NS |
| Liver cirrhosis | | 0 (0%) | | 3 (0.3%) | NS | NS |
| Elevated pulmonary artery hypertension | | 23 (37.1%) | | 319 (34.7%) | 1.1 (0.7-1.9) | 0.7 |
| Chronic lung disease | | 12 (19.4%) | | 117 (12.7%) | 1.6 (0.9-3.2) | 0.14 |
| Active smoking | | 6 (9.7%) | | 125 (13.6%) | 0.7 (0.3-1.6) | 0.38 |
| Ex-smoker | | 19 (30.6%) | | 290 (31.5%) | 1.0 (0.6-1.7) | 0.89 |
| Obstructive sleep apnea | | 5 (8.1%) | | 96 (10.4%) | 0.8 (0.3-1.9) | 0.55 |
| CPAP | | 4 (6.5%) | | 67 (7.3%) | 0.9 (0.3-2.5) | 0.81 |
| Any malignancy | | 10 (16.1%) | | 105 (11.4%) | 1.5 (0.7-3.0) | 0.27 |
| Active malignancy | | 0 (0%) | | 10 (1.1%) | NS | NS |
| Autoimmune disease | | 7 (11.3%) | | 135 (14.7%) | 0.7 (0.3-1.7) | 0.47 |
| Connective tissue disease | | 1 (1.6%) | | 11 (1.2%) | 1.4 (0.2-10.7) | 0.77 |
| Skin disease | | 2 (3.2%) | | 67 (7.3%) | 0.4 (0.1-1.8) | 0.24 |
| Critical preoperative state | | 1 (1.6%) | | 4 (0.4%) | 3.8 (0.4-34.1) | 0.24 |
| ***Type of procedure:*** | |  | |  |  |  |
| AVR | | 19 (30.6%) | | 285 (31.0%) | 1.0 (0.6-1.7) | 0.96 |
| CABG | | 43 (69.4%) | | 512 (55.7%) | 1.8 (1.0-3.1) | 0.037 |
| Any mitral valve procedure | | 4 (6.5%) | | 162 (17.6%) | 0.3 (0.1-0.9) | 0.031 |
| MVP | | 3 (4.8%) | | 108 (11.7%) | 0.4 (0.1-1.2) | 0.11 |
| MVR | | 1 (1.6%) | | 55 (6.0%) | 0.3 (0.0-1.9) | 0.18 |
| Ascending aorta | | 4 (6.5%) | | 100 (10.9%) | 0.6 (0.2-1.6) | 0.28 |
| David procedure | | 2 (3.2%) | | 22 (2.4%) | 1.4 (0.3-5.9) | 0.68 |
| Bentall-DeBono procedure | | 5 (8.1%) | | 49 (5.3%) | 1.6 (0.6-4.1) | 0.36 |
| Maze procedure | | 1 (1.6%) | | 24 (2.6%) | 0.6 (0.1-4.6) | 0.63 |
| Pericardiectomy | | 0 (0%) | | 5 (0.5%) | NS | NS |
| LAA closure | | 8 (12.9%) | | 135 (14.7%) | 0.9 (0.4-1.9) | 0.7 |
| Other | | 0 (0%) | | 36 (3.9%) | NS | NS |
| ***Operation specifics:*** | |  | |  |  |  |
| CPB | | 51 (82.3%) | | 773 (84.0%) | 0.9 (0.5-1.7) | 0.72 |
| EuroSCORE II (%) | | 1.91 (1.37-2.98) | | 1.48 (0.97-2.56) | 1.1 (1.0 -1.2) | 0.19 |
| ACS | | 30 (48.4%) | | 275 (29.9%) | 1.8 (1.0-3.3) | 0.038 |
| Urgent, emergency, or salvage | | 14 (22.6%) | | 171 (18.6%) | 1.3 (0.7-2.4) | 0.44 |
| Operation length (min) | | 236 (201-271) | | 228 (197-267) | 1.0 (1.0-1.0) per 10 units | 0.92 |
| Aortic cross-clamping time (min) | | 99 (26) | | 90 (76-111) | 1.0 (1.0-1.1) per 10 units | 0.41 |
| CPB time (min) | | 122 (107-148) | | 119 (102-145) | 1.0 (1.0-1.1) per 10 units | 0.77 |
| Antegrade cardioplegia | | 48 (77.4%) | | 700 (76.0%) | 1.0 (0.5-1.9) | 0.98 |
| Retrograde cardioplegia | | 14 (22.3%) | | 186 (20.2%) | 1.1 (0.6-2.1) | 0.67 |
| Delayed ventilation | | 0 (0%) | | 77 (8.4%) | NS | NS |
| Duration of mechanical ventilation (hours) | | 7 (5-10) | | 6 (4-10) | 1.0 (1.0-1.0) | 0.25 |
| ***ICU:*** | |  | |  |  |  |
| Length of ICU stay (hours) | | 22 (22-24) | | 23 (22-25) | 1.0 (0.9-1.0) | 0.017 |
| Total intravenous fluids (mL) | | 3007 (880) | | 2669 (2192-3296) | 1.0 (1.0-1.0) per 10 units | 0.87 |
| Chest drain output (mL/12 h) | | 409 (310-520) | | 385 (290-540) | 1.0 (1.0-1.0) per 10 units | 0.44 |
| Diuresis (mL/12 h) | | 1360 (954-1853) | | 1450 (1041-1969) | 1.0 (1.0-1.0) per 10 units | 0.21 |
| Noradrenaline infusion | | 62 (100.0%) | | 903 (99.4%) | NS | NS |
| Noradrenaline total dose (mg) | | 4.8 (3.1-9.2) | | 3.3 (1.2-8.8) | 1.0 (1.0-1.0) | 0.3 |
| Adrenaline infusion | | 3 (4.8%) | | 31 (3.4%) | 1.5 (0.4-4.9) | 0.55 |
| ***Blood products:*** | | 35 (56.5%) | | 414 (45.0%) | 1.6 (0.9-2.7) | 0.08 |
| RBC transfusion | | 30 (48.4%) | | 304 (33.0%) | 1.9 (1.1-3.2) | 0.015 |
| RBC units/patient | | 2.0 (1.8-4.3) | | 2.0 (2.0-4.0) | 0.9 (0.8-1.1) | 0.36 |
| Fresh frozen plasma | | 11 (17.7%) | | 193 (21.0%) | 0.8 (0.4-1.6) | 0.54 |
| Platelets | | 8 (12.9%) | | 206 (22.4%) | 0.5 (0.2-1.1) | 0.09 |
| Lowest Hb postoperatively (g/L) | | 111 (13) | | 114 (102-125) | 0.9 (0.8-1.1) per 10 units | 0.29 |
| Lowest Hb during the 1^st^ postoperative week (g/L) | | 85 (78-95) | | 93 (83-104) | 0.6 (0.4-0.8) per 10 units | <0.001 |
| Values denote n (%), mean ± standard deviation, or median (25th - 75th percentile), as appropriate. Binary logistic regression: Odds ratio with 95% confidence interval. Abbreviations: ACS: acute coronary syndrome; AVR: aortic valve replacement; BMI: body mass index; CABG: coronary artery bypass grafting; CPAP: continuous positive airway pressure; CPB: cardiopulmonary bypass; eGFR: estimated glomerular filtration rate; Hb: hemoglobin; ICU: intensive care unit; LAA: left atrial appendix; MVP: mitral valvuloplasty; MVR: mitral valve replacement; NS: not significant; PCI: percutaneous coronary intervention; RBC: packed red blood cell; SIRS: systemic inflammatory response syndrome; TAVI: transcatheter aortic valve implantation. Prior cardiac procedures: PCI, CABG, aortic or mitral valve prosthesis, or TAVI. Autoimmune disease: composite variable for hyperthyroidism, hypothyroidism, inflammatory bowel disease, rheumatoid arthritis, psoriatic arthropathy, and systemic lupus erythematosus. Connective tissue disease: composite variable for Marfan syndrome, Ehlers-Danlos syndrome, Turner syndrome, and Loeys-Dietz syndrome. Preoperative eGFR was calculated using CKD-EPI equation. Delayed ventilation defined longer than 24 hours. | | | | | | |
|  |  |  |  |  |  |  |

**Supplementary Table 3.** Preoperative medication in patients with and without prolonged SIRS.

|  | **Prolonged SIRS**  **n = 62** | **No prolonged SIRS**  **n = 920** | **OR (95% CI)** | **P-value** |
| --- | --- | --- | --- | --- |
| ***Rhythm control:*** |  |  |  |  |
| Amiodarone | 0 (0%) | 5 (0.5%) | NS | NS |
| Beta blockers | 43 (69.4%) | 596 (64.8%) | 1.2 (0.7-2.1) | 0.47 |
| Digoxin | 0 (0%) | 24 (2.6%) | NS | NS |
| Sotalolol | 0 (0%) | 0 (0%) | NS | NS |
| Verapamil | 0 (0%) | 2 (0.2%) | NS | NS |
| ***Treatment for hypertension:*** |  |  |  |  |
| ACE or ARB | 39 (62.9%) | 577 (62.7 %) | 1.0 (0.6-1.7) | 0.98 |
| CCBs | 24 (38.7%) | 245 (26.6%) | 1.7 (1.0-3.0) | 0.041 |
| Diuretics | 18 (29.0%) | 225 (24.5%) | 1.3 (0.7-2.3) | 0.42 |
| ***Treatment for diabetes:*** |  |  |  |  |
| Insulin | 11 (17.7%) | 100 (10.9%) | 1.8 (0.9-3.5) | 0.1 |
| Oral diabetic medicine | 14 (22.6%) | 190 (20.7%) | 1.1 (0.6-2.1) | 0.72 |
| SGLT2 inhibitor | 3 (4.8%) | 22 (2.4%) | 2.1 (0.6-7.1) | 0.25 |
| ***Antithrombotics:*** |  |  |  |  |
| ASA | 37 (59.7%) | 478 (52.0%) | 1.4 (0.8-2.3) | 0.24 |
| ADP receptor inhibitor | 4 (6.5%) | 57 (6.2%) | 1.0 (0.4-3.0) | 0.94 |
| DOAC | 9 (14.5%) | 96 (10.4%) | 1.5 (0.7-3.0) | 0.32 |
| LMWH | 14 (22.6%) | 136 (14.8%) | 1.7 (0.9-3.1) | 0.1 |
| Warfarin | 7 (11.3%) | 94 (10.2%) | 1.1 (0.5-2.5) | 0.79 |
| ***Treatment for dyslipidemia:*** |  |  |  |  |
| Any | 0 (0%) | 7 (0.9%) | NS | NS |
| Etzetimibe | 0 (0%) | 14 (1.5%) | NS | NS |
| Statin | 47 (75.8%) | 620 (67.4%) | 1.5 (0.8-2.8) | 0.17 |
| ***Antianginal:*** |  |  |  |  |
| ISMN | 11 (17.7%) | 173 (18.8%) | 0.9 (0.5-1.8) | 0.84 |
| Values denote n (%), mean ± standard deviation, or median (25th - 75th percentile), as appropriate. Binary logistic regression: Odds ratio with 95% confidence interval. Diuretics: hydrochlorothiazide, loop diuretics, spironolactone. ACE: angiotensin-converting enzyme; ADP: adenosine diphosphate; Abbreviations: ASA: acetylsalicylic acid; ARB: angiotensin II receptor; CCBs: calcium channel blockers; DOAC: direct oral anticoagulants; ISMN: isosorbide mononitrate; LMWH: low-molecular-weight heparin; NS: not significant; SIRS: systemic inflammatory response syndrome; SGLT-2: sodium-glucose cotransporter-2. | | | | |

**Supplementary Table 4.**

|  | **Prolonged SIRS**  **n = 62** | **No prolonged SIRS**  **n = 920** | **p-value** |
| --- | --- | --- | --- |
| ***Highest cTnT levels:*** |  |  |  |
| During operation (ng/L) | 655 (397-1249) | 590 (355-968) | 0.18 |
| The first postoperative day (ng/L) | 512 (240-900) | 413 (266-688) | 0.22 |
| During the first postoperative week (ng/L) | 687 (420-1282) | 601 (360-1004) | 0.09 |

Values denote n (%), mean ± standard deviation, or median (25th - 75th percentile), as appropriate. Abbreviations: cTnT: cardiac troponin T; SIRS: systemic inflammatory response syndrome.

**Supplementary Table 5.**

|  | OR | p-value |
| --- | --- | --- |
| Preoperative eGFR (ml/min/1.73 m2) | 0.985 (0.970-0.999) | 0.040 |
| Treatment for type 1 diabetes | 3.292 (1.312-8.257) | 0.011 |
| Treatment for hypertension | 2.382 (1.193-4.753) | 0.014 |
| Operation for ACS | 1.849 (1.036-3.301) | 0.038 |
| RBC transfusion | 1.900 (1.133-3.185) | 0.015 |
| CRP on the first POD | 1.017 (1.007-1.026) | <0.001 |
| Binary logistic regression: Odds ratio with 95% confidence interval. Abbreviations: ACS: acute coronary syndrome**;** CRP: C-reactive protein; eGFR: estimated glomerular filtration rate; POD: post-operative day; RBC: packed red blood cell**.** | | |

**Supplementary Table 6.**

|  | OR | p-value |
| --- | --- | --- |
| Male sex | 1.548 (1.029-2.331) | 0.036 |
| Preoperative eGFR (ml/min/1.73 m2) | 1.019 (1.008-1.030) | <0.001 |
| Operation CABG | 0.407 (0.278-0.596) | <0.001 |
| Operation AVR | 2.144 (1.462-3.145) | <0.001 |
| Operation LAA closure | 3.615 (1.739-7.516) | <0.001 |
| Operation mitral valve | 2.338 (1.455-3.759) | <0.001 |
| Operation ascending aorta | 1.777 (1.017-3.103) | 0.043 |
| CPB | 2.315 (1.268-4.237) | 0.006 |
| Prolonged SIRS | 2.058 (1.049-4.037) | 0.036 |
| Binary logistic regression: Odds ratio with 95% confidence interval. Abbreviations: AVR: aortic valve replacement; CABG: coronary artery bypass grafting; CPB: cardiopulmonary bypass; eGFR: estimated glomerular filtration rate; LAA: left atrial appendix. | | |

**Supplementary Figure 1**


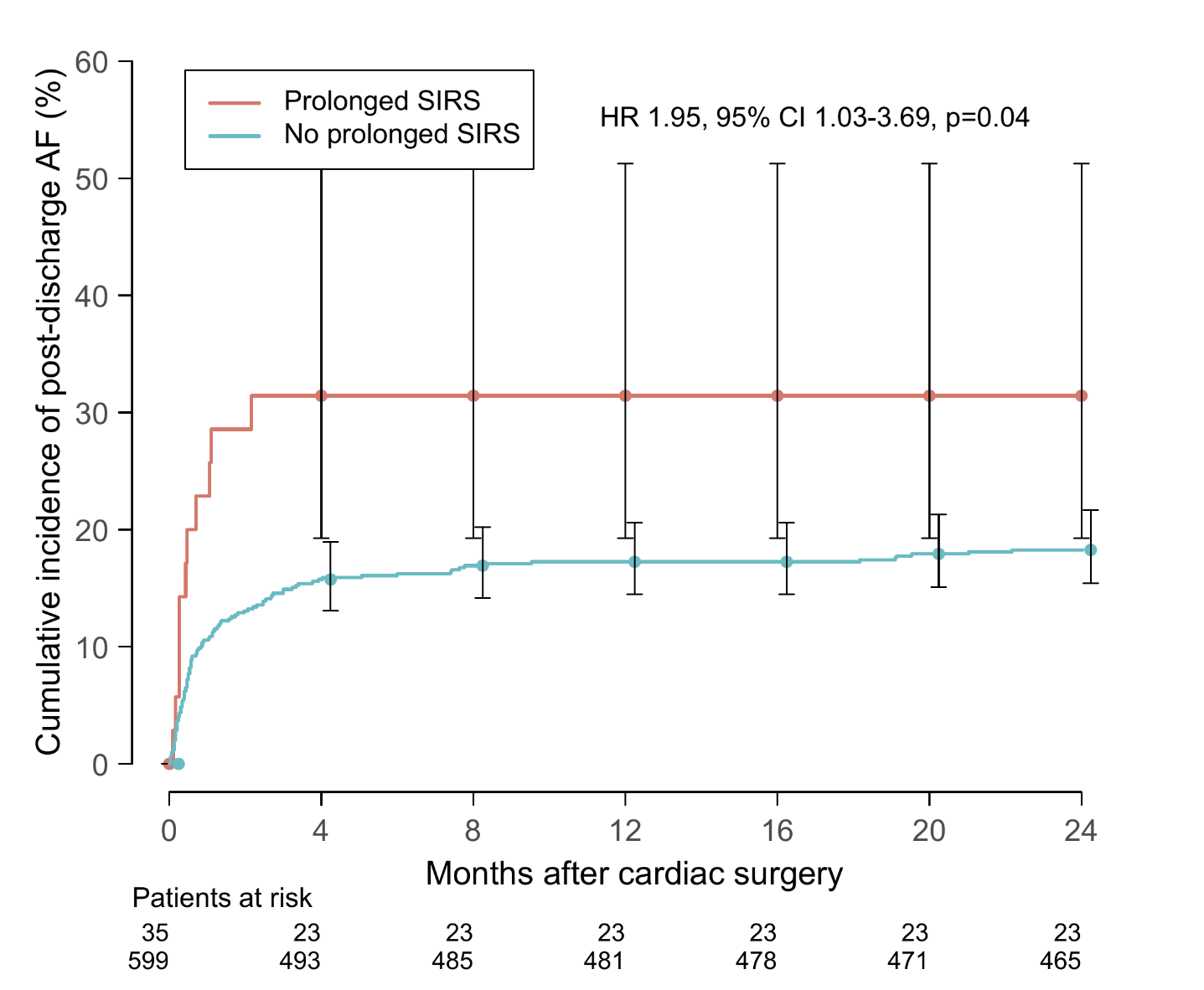

Supplement: ivag081_Supplementary_Data [file ivag081_supplementary_data.docx]
